# Supplementary material for: Development of a computer-aided design software for the quantitative evaluation of aesthetic damage
Source: PLoS One. 2019 Dec 18;14(12):e0226322. doi: 10.1371/journal.pone.0226322 (PMC6919621; doi:10.1371/journal.pone.0226322)
Supplement: S1 Code site — 1. (ZIP) [file pone.0226322.s001.zip › [EESC_jr][Projeto]Relatorio_07_21/vendors/select2/docs/_includes/options-old/dropdown.html]

# Dropdown

Select2 allows you to change the way that the dropdown works, allowing you
to do anything from attach it to a different location in the document or
add a search box.

## Attached to body

By default, Select2 will attach the dropdown to the end of the body and
will absolutely position it to appear below the selection container.

Key
:   `dropdownParent`

Value
:   jQuery element or DOM node

---

Adapter
:   `DropdownAdapter`

Decorator
:   `AttachBody`

**Heads up!**
This will cause DOM events to be raised outside of the standard
Select2 DOM container. This can cause issues with
third-party components such as modals.

When the dropdown is attached to the body, you are not limited to just
displaying the dropdown below the container. Select2 will display above
the container if there is not enough space below the container, but there
is enough space above it. You are also not limited to displaying the
dropdown within the parent container, which means Select2 will render
correctly inside of modals and other small containers.

## Attached below the container

Select2 can place the dropdown directly after the selection container, so
it will appear in the same location within the DOM as the rest of Select2.

Adapter
:   `DropdownAdapter`

Decorator
:   `AttachContainer`

**Check your build.** This module is only included in the
full builds of
Select2.

**Harvest Chosen
migrators!** 
If you are migrating to Select2 from Chosen, this option will cause
Select2 to position the dropdown in a similar way.

## Search

Users can filter down the results by typing a search term into a box that
is displayed at the top of the dropdown.

Adapter
:   `DropdownAdapter`

Decorator
:   `DropdownSearch`

A search box is added to the top of the dropdown automatically for select
boxes where only a single option can be selected.

### Minimum search term length to filter results

Sometimes when working with large data sets, it is more efficient to start
filtering the results when the search term is a certain length. This is
very common when working with remote data sets, as allows for only
significant search terms to be used.

Key
:   `minimumInputLength`

Value
:   integer

Adapter
:   `DataAdapter`

Decorator
:   `MinimumInputLength`

### Maximum search term length to filter results

In some cases, search terms need to be limited to a certain range. Select2
allows you to limit the length of the search term such that it does not
exceed a certain length.

Key
:   `maximumInputLength`

Value
:   integer

Adapter
:   `DataAdapter`

Decorator
:   `MaximumInputLength`

### Minimum results to display the search box

When working with smaller data sets, the search box can take up more space
that is necessary, as there are not enough results for filtering to be
effective. Select2 allows you to only display the search box when the
number of search results reaches a certain threshold.

Key
:   `minimumResultsForSearch`

Value
:   integer

Adapter
:   `DropdownAdapter`

Decorator
:   `MinimumResultsForSearch`

## Select the highlighted option on close

When users close the dropdown, the last highlighted option can be
automatically selected. This is commonly used in combination with
tagging and placeholders
and other situations where the user is required to select an option, or
they need to be able to quickly select multiple options.

Adapter
:   `ResultsAdapter`

Decorator
:   `SelectOnClose`

## Close the dropdown when a result is selected

Select2 will automatically close the dropdown when an element is selected,
similar to what is done with a normal select box. This behavior can be
changed though to keep the dropdown open when results are selected,
allowing for multiple options to be selected quickly.

Key
:   `closeOnSelect`

Default
:   `true`

Adapter
:   `DropdownAdapter`

Decorator
:   `CloseOnSelect`

If this decorator is not used (or `closeOnSelect` is set to
`false`), the dropdown will not automatically close when a
result is selected. The dropdown will also never close if the
`ctrl` key is held down when the result is selected.
